# Asymmetrical distribution of the transcriptionally competent NORs in mitosis

**DOI:** 10.1016/j.jsb.2008.04.002

**Published:** 2008-07

**Authors:** Markéta Kalmárová, Lubomír Kováčik, Alexey Popov, Sánchez Pilar Testillano, Evgeny Smirnov

**Affiliations:** a Institute of Cellular Biology and Pathology, First Faculty of Medicine, Charles University in Prague, Albertov 4, 128 01 Prague 2, Czech Republic; b Department of Cell Biology, Institute of Physiology, Academy of Sciences of the Czech Republic v.v.i., Albertov 4, 128 01 Prague 2, Czech Republic; c Center of Biological Researches (CIB), CSIC, Ramiro de Maeztu 9, E-28040 Madrid, Spain

**Keywords:** NORs, UBF, Mitosis, Asymmetry, 4D imaging, GFP-UBF

## Abstract

Ribosomal genes are organized in clusters termed Nucleolus Organizer Regions (NORs). Essential components of the RNA polymerase I transcription machinery, including Upstream Binding Factor (UBF), can be detected on some NORs during mitosis; these NORs, termed competent, are believed to be transcriptionally active during interphase. In cultured mammalian cycling cells, the number of competent NORs, and their distribution among the different chromosomes, does not vary significantly in the sequential cell cycles. In this work we investigate whether this stable state is achieved by equal distribution of competent NORs during cell division. To answer this question we first studied the state of NORs in telophase HeLa and LEP cells. In both cell lines we found a small but significant difference between the emerging daughter cells in the number of UBF-loaded NORs. To reveal the cause of this difference, we followed the fate of individual NOR using HeLa derived cell line stably expressing UBF-GFP. We demonstrated that some NORs in metaphase are “asymmetrical”, i.e. they lack the signal of competence on one of the sister chromatids. Regular presence of such NORs can account for the difference in the number of competent NORs obtained by the daughter cells emerging in mitosis.

## 1 Introduction

Ribosomal genes coding 5.8S, 18S and 28S rRNA are organized in clusters which can be identified in mitotic chromosomes and are called Nucleolus Organizer Regions (NORs). In normal human cells these regions are situated on the short arms of the acrocentric chromosomes (Henderson et al., 1972; Long and Dawid, 1980; Puvion-Dutilleul et al., 1991; Raška, 2003; Raška et al., 2006). Although rDNA transcription is efficiently blocked from prophase to late anaphase, at least some subunits of RNA polymerase I (pol I) along with its main transcription factors, the Upstream Binding Factor (UBF) and promoter selectivity complex (SL1), can be detected on certain NORs even in metaphase (Babu and Verma, 1985; Weisenberger and Scheer, 1995; Jordan et al., 1996; Roussel et al., 1996; Gebrane-Younes et al., 1997; Sirri et al., 1999; Leung et al., 2004; Prieto and McStay, 2005). Such NORs, termed “transcriptionally competent” or “competent” (Dousset et al., 2000; Savino et al., 2001), can be also visualized on the chromosome spreads by silver staining (Howell and Black, 1980). It is generally accepted that competent NORs are transcribed, while the others, “non-competent” NORs, remain silent throughout the previous interphase (Weisenberger and Scheer, 1995; Roussel et al., 1996; Gebrane-Younes et al., 1997). UBF, the most abundant component of the competent NORs, serves apparently as an architectural element responsible for transcriptionally favorable state of chromatin (O’Sullivan et al., 2002; Mais et al., 2005; Prieto and McStay, 2007).

Studies on mitotic chromosomal spreads in cultured cycling cells show that the total number of competent NORs per cell, and frequency with which they appear on certain chromosomes, do not vary significantly in the sequential cell cycles (Heliot et al., 2000; Smirnov et al., 2006). Thus, for example, one of three chromosomes 14 in human derived transformed HeLa cells regularly carries competent NOR, while the NORs belonging to the other two chromosomes remain silent; all chromosomes 15 usually carried competent NORs (Smirnov et al., 2006). Equal distribution of competent NORs between the daughter cells emerging in mitosis seems to be the easiest way to achieve such stable propagation, for in this case there is no need to change the NOR competence status of chromosomes in the course of interphase. But this idea was not experimentally verified. On the other hand, it was shown that the number of nucleoli or intranucleolar silver stained foci is not heritable through mitosis (Kalmárová et al., 2008; Leung et al., 2004; Smetana et al., 1999).

Here we compared the state of NORs in daughter cells emerging at the end of mitosis. Using immunocytochemistry, we observed, in both transformed HeLa and diploid LEP cells, a small but significant asymmetry in the distribution of competent NORs during cell division. Following the segregation of competent NORs during mitosis *in vivo* in a HeLa derived cell line stably expressing UBF-GFP (HeLa-UBF), we found that chromosomes with asymmetrical NORs, in which only one of sister chromatids carries the signal of competence, are the main source of the observed mitotic asymmetry.

## 2 Materials and methods

### 2.1 Cell culture

We used HeLa, aneuploid cells that have stable karyotype without considerable variations (Macville et al., 1999; Smirnov et al., 2006), and LEP, human diploid fibroblasts derived from embryonic lung. The cells were cultivated in flasks or on coverslips at 37 °C in Dulbecco’s modified Eagle’s medium (DMEM, Sigma, USA) containing 10% fetal calf serum, 1% glutamine, 0.1% gentamycin and 0.85 g/l NaHCO_3_ in atmosphere supplemented with 5% CO_2_.

### 2.2 In situ hybridization

Biotin-labeled rDNA probe was used for visualization of NORs. The probe was prepared from a pB plasmid construct (Erickson et al., 1981), kindly donated by James Sylvester (Nemours Children’s Clinic Research, Orlando, FL). The pB probe contains the promoter, the external transcribed spacer, and the 5‘ end of the 18S subfragment. The probe was labeled by biotin using nick-translation kit BIONICK Labeling System (Gibco-BRL, Invitrogen) according to the manufacturer’s instructions. The rDNA probe was stored in hybridization mixture containing 25 ng of probe, 0.5 mg/ml sonicated salmon sperm DNA, 50% deionized formamide, 2× SSC and 10% dextran sulfate at −20 °C.

For detection of NORs, the cells on coverslips were fixed in methanol:acetic acid (3:1), rinsed in 2× SSC, pH 7, and incubated with 100 μg/ml RNAse A (Roche) for 1 h at 37 °C, gradually dehydrated in ice-cold 70, 80 and 96% ethanol, and air-dried. The denaturation of the chromosomal DNA was performed in 70% deionized formamide in 2× SSC, pH 7, at 72 °C for 3 min. The probe was denatured at 70 °C for 8 min. Hybridization ran overnight at 37 °C in moisture chamber. After hybridization the cells were washed 15 min in 50% formamide in 2× SSC, pH 7, at 43 °C; 8 min in 0.1% Tween 20 in 2× SSC at 43 °C; and 3 × 4 min in 0.1% Igepal (ICN Biomedicals, Inc) in 4× SSC. Biotinylated rDNA probe was labeled after FISH with monoclonal rabbit anti-biotin antibodies (Enzo, Roche). Secondary anti-mouse and anti-rabbit antibodies (Jackson ImmunoResearch Laboratories) were conjugated with Cy3 or FITC. Coverslips were mounted in Mowiol and viewed using Leica SP5 confocal microscope.

### 2.3 UBF and pol I immunocytochemistry

Fixed cells were rinsed in PBS and fixed in 2% paraformaldehyde for 10 min at RT, and permeabilized with 0.2% Triton X-100. Primary antibody against human UBF and pol I was kindly provided by U. Scheer, (Biocenter of the University of Wurzburg). We also used monoclonal (mouse) anti-UBF antibody (Santa Cruz Biotechnology, Inc.), which binds human UBF. Secondary anti-human antibodies were labeled with Cy3 or FITC (Jackson ImmunoResearch Laboratories). Coverslips were mounted in Mowiol. Specially, for the cells fixed in methanol/acetic acid, incubation with UBF antibody was performed in moisture chamber for 1 h at 37 °C.

NORs were counted using 3D confocal images on SP5 microscope (Leica). The same numbers of UBF signals in the cells were obtained using Olympus AX70 Provis equipped with the Photometrics CCD camera that provides higher sensitivity. The data were compared with random pairing model in which appearance of the pair of cells with *i* and *j* NORs was calculated as product of the experimentally found frequencies of the cells with *i* and *j* NORs (random pairing model).

### 2.4 4D imaging of competent NORs in living cells

For in vivo study of the competent NORs we used HeLa-GFP cell line steady expressing GFP-tagged UBF, kindly provided by A. Lamond (Division of Gene Regulation and Expression, School of Life Sciences, Wellcome Trust Biocentre, University of Dundee, Scotland UK). The cells were seeded on glass bottom dishes (Mat Teck, USA). 4D imaging was performed on confocal microscope SP5 using argon laser with objective HCX PL APO lambda blue 63 × 1.4. As the studied cells most frequently contained 8–10 competent NORs, we selected for the observation 50 cells with such numbers of GFP signals. A 3D image stack of 25–30 optical sections, spanning the entire cell volume was recorded at each time point. Low light imaging conditions and resonance scanner regime were chosen to ensure that cells were able to progress from metaphase to the end of telophase. We chose the period of 1 min between the sequential stacks, which allowed to follow the movements of individual NORs without impairing the ability of the cell to divide.

## 3 Results

We observed on mitotic chromosomes 6–12 competent NORs (in average 9) in HeLa and 6–10 competent NORs (in average 8) in LEP cells, using antibodies against human UBF, mouse UBF and pol I (each of these antibodies can be used for detecting competent NORs). These data were in agreement with our previous study (Smirnov et al., 2006).

**Fig. 1:**
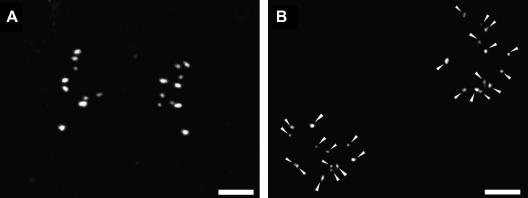
Asymmetrical distribution of the competent NORs (A) and symmetrical distribution of NORs (B) between the daughter cells in telophase. (A) Competent NORs are labeled with antibody against UBF. There are 9 UBF signals in the left cell, and 10 in the right. The left cell is short of one signal. (B) Both emerging cells have 13 rDNA hybridization signals, which is typical number of NORs in HeLa (Smirnov et al., 2006). Scale bar: 5 μm.

In metaphase cells the competent NORs appeared mostly as double dots representing pairs of half-NORs. There were also single signals, small or large, in the last case they could be elongated. Detecting UBF signals in telophase, we found that the competent NORs were distributed among the daughter cells with a certain asymmetry: the emerging daughter cells got equal numbers of competent NORs only in about a quarter of telophases (Table 1). In extreme cases, the difference in number of UBF signals reached 5 or 6, but most frequently, the daughter cells differed by one competent NOR, and the expected difference between the daughter cells was about 1 in both HeLa and LEP cells (columns 2 and 8; Fig. 1A). The observed asymmetry was close to, though still lower than that predicted by the model in which cells were randomly paired (column 2). To check our result, we compared the mean values of the competent NORs counted on metaphase chromosomal spreads and in telophase cells, and found no significant difference between these values (data not shown), indicating that occasional clumping of UBF signals in the cells could not have an essential impact on our result.

Since asymmetry in distribution of the competent NORs between the daughter cells might result from asymmetrical distribution of NORs, we also performed rDNA hybridization and detected the whole pool of NORs in metaphase and telophase. In HeLa cells the number of NORs counted on the preparations of spread mitotic chromosomes, varied from 10 to 15 (in average 13). In LEP cells, 10 NORs could be observed in 99 % cases, which agreed with our previous data (Smirnov et al., 2006). In telophase LEP cells we usually found a perfect symmetry: each of the daughter cells contained 10 NORs. In HeLa cells the number of rDNA signals varied from cell to cell as in metaphase, with mean value 13. But in most cases (64%) the daughter cells had the same numbers of NORs (Fig. 1B), whereas the random pairing model, in which the daughter cells are regarded as independent (see Section 2), predicted 22% (Table 2, compare with the Table 1 that shows predominantly asymmetrical distribution of competent NORs between the daughter cells). Thus, in both HeLa and LEP cells, the NORs were divided between the daughter cells in mitosis rather equally. In other words, the asymmetry in distribution of the NORs, in both HeLa and LEP cells, was not as significant as to account for the asymmetry in distribution of the competent, UBF-positive, NORs.

**Fig. 2:**
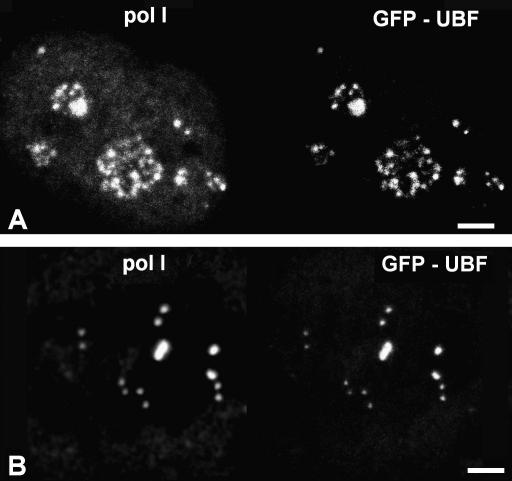
Colocalization of GFP-UBF and pol I in the HeLa GFP cell line in interphase and mitotic cells. Since pol I co-localizes with UBF (data not shown) it can be used for mapping the endogenous UBF. A, interphase; B, metaphase. Scale bar: 5 μm.

**Fig. 3:**
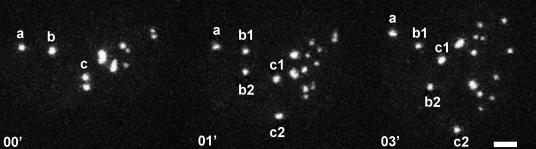
Presence of some special NORs can account for the difference in the numbers of competent NORs obtained by the daughter cells in mitosis. Three NORs of different types are selected for comparison: a, single “asymmetrical”; b, single “symmetrical”; c, double, the most common type of NOR. Time course 0.0 min—metaphase. NORs a and b are seen as single dots, but c is represented by a double dot. Time course 01 min—beginning of anaphase. NORs b and c are divided into b1,2 and c1,2. NOR a remains single, is marked as a1. Time course 0.2 min—end of anaphase. NORs c1 and c2, as well as b1 and b2, are moving to the opposite poles of mitosis. NOR a1 has no counterpart. As a result, one of the two emerging daughter cells (top) acquires nine competent NORs, the other (bottom) only eight. Scale bar: 5 μm.

Next we questioned whether the observed asymmetry between the daughter cells appears already at the segregation of daughter chromatids (together with their NORs) in the beginning of anaphase, or some NORs can acquire or lose UBF loading later in mitosis. To answer this we followed the dynamics of competent NORs from metaphase to the end of telophase in the HeLa derived cell line expressing UBF-GFP. In the mitotic HeLa-GFP cells, the GFP signals coincided with NORs stained with antibodies against UBF and Pol I (Fig. 2A and B), and the number of competent NORs equaled the number of GFP-positive foci. Analysis of the 4D images showed that at the beginning of anaphase, the half-NORs belonging to all double NORs, as well as to some single NORs, were separated and subsequently distributed between the emerging daughter cells (Fig. 3) But in a fraction of single NORs, no segregation of half-NORs was observed. One to three of such NORs were found in about 50% of the studied HeLa-UBF cells. These NORs moved towards one of the mitotic poles, thus producing an asymmetrical state in respect to the number of competent NORs. After segregation of all half-NORs, which was not simultaneous, but occurred in some NORs 1–2 min later than in the rest, the number of visible GFP signals did not change until the termination of cytokinesis. In early G1, the UBF-positive signals expanded, often divided in two or more foci, and began to fuse, so that individual NORs could not be identified in the newly formed cell nucleoli.

Thus the asymmetry in distribution of the competent NORs between the daughter cells appears at the beginning of anaphase and persists until the end of mitosis.

## 4 Discussion

Our study shows that both HeLa and LEP cells regularly exhibit an asymmetry of mitosis: the daughter cells receive different numbers of the transcriptionally competent NORs. This asymmetry can be observed at the beginning of anaphase, which implies that it should exist in a hidden form already in metaphase. Different types of competent NORs can be found on preparations of metaphase chromosome spreads, stained with silver or antibody against UBF (Smirnov et al., 2006). Some of them are seen as double dots, others as single dots. In most of the single NORs, the signals of transcription competence are situated on both sister chromatids but very close to each other, as was shown in an electron tomography study (Heliot et al., 1997). In another, relatively rare group of NORs, the silver signal appears as a single small dot, and some authors speculated that it belongs to only one of the chromatids (Heliot et al., 2000), but this could not be proven by the method employed in the cited work.

In the present study, using HeLa-UBF cell line, we could follow the fate of individual competent NORs throughout the mitosis. In about a half of the dividing cells we observed single UBF-positive signals that apparently belonged to only one sister chromatid, for they did not split in anaphase, but were drawn towards one pole of the cell division, which could lead to unequal distribution of the competent NORs between the emerging daughter cells. Namely, when the number of half-NORs in metaphase was odd, they could not be equally divided between two daughter cells.

We thus demonstrated the presence of “really single”, or asymmetrical, NORs which are at least partially responsible for the observed asymmetry in mitosis. In HeLa cells the number of NORs is variable, and this variability should also contribute to the mitotic asymmetry, but only to a very limited extent, because NORs are symmetrically distributed between the daughter cells in most cases (Table 2, Fig. 1B). Importantly, following the 3D movements of individual UBF signals after metaphase *in vivo*, we demonstrated that NORs do not change their competence in the course of mitosis, and so there is no switch between symmetrical and asymmetrical state.

As the average number of competent NORs per cell remains stable in the cycling cell population (Smirnov et al., 2006), it is not quite clear how this stable state is sustained in HeLa and LEP cells, in which emerging daughter cells regularly encompass different numbers of competent NORs. It has been found (Heliot et al., 2000; Smirnov et al., 2006) that competent NORs are non-randomly distributed among the different chromosomes, so that some NORs become competent more frequently than others. As the competent NORs, visualized after premature chromosome condensation with calyculin A, remain single throughout G1, and double NORs first appear in the S phase (Smirnov et al., 2006), we hypothesize that the transcription competence of NORs is re-established in association with their replication, and loading of the newly synthesized chromatids with UBF, as well as other components of the transcription machinery, depends on the specific type of chromosomes /NORs. For instance, in HeLa cells all homologs of the chromosome 15 will carry signal of competence with high probability, whereas for the chromosome 14 such probability is much lower. As a result the average number of competent NORs remains constant in the sequential cell generations.

Our data imply that different numbers of NORs should be active in the daughter cells. This slight asymmetry very likely leads to a slight asymmetry in the level of transcription activity since the very onset of transcription, i.e. from the end of anaphase. However, according to our data, the asymmetry in respect to the competent NORs is not corrected at least until the end of telophase. We suppose that such correction should take place later, during S phase, when most of the competent NORs (but not all) become duplicated. Although we still cannot exclude that the number of competent NORs may change in G1, S phase is crucial for establishing competence status of NORs. On the other hand, transcription of ribosomal genes can be efficiently regulated without changing the number of competent NORs (Grummt, 2007; Russell and Zomerdijk, 2005; Moss, 2004). Therefore, we do not regard the correction of the described asymmetry as a direct compensatory response to the shift in rRNA production.

To conclude, we have described a small but significant asymmetry in distribution of the transcriptionally competent NORs between the emerging daughter cells in mitosis. Presence of chromosomes with asymmetrical NORs in which only one of sister chromatids carries the signal of competence can account for this asymmetry.

## Figures and Tables

**Table 1:** Asymmetry in the distribution of the transcriptionally competent NORs between the two emerging daughter cells in telophase

HeLa									
	0	1	2	3	4	5	6	ED	NED
Model	0.21	0.30	0.24	0.15	0.07	0.03	0.01	1.72	
Experiment	0.23	0.44	0.22	0.10	0.01	0	0	1.22	0.14
									
LEP									

	0	1	2	3	4	ED	NED		
Model	0.25	0.41	0.23	0.09	0.02	1.22			
Experiment	0.24	0.62	0.14	0	0	0.90	0.11		

The table shows frequencies *f*_i_ with which the number of UBF signals in daughter cells differs by 0 (symmetrical distribution), 1, 2, and so on. Experimental data, obtained from 200 cells are compared with random pairing model (see Section 2.3). Thus, in both cell lines, less than a quarter of the cell pairs were symmetrical with regard to the number of competent NORs. Most frequently the daughter cells differed by one competent NOR. ED stands for “expected difference” in the number of signals between the daughter cells; it was computed as Σ*f*_i_*d*_i_, where *d*i is the difference in the number of signals between the daughter cells, and *f*i is the corresponding frequency. NED is the “normalized expected difference”, calculated as ED/n, i.e. the value of expected difference divided by the mean number of the signals in given cell population (9 in HeLa, and 8 in LEP cells). NED, which is actually a measure of asymmetry per 1 signal, took on close values in HeLa and LEP cells.

**Table 2:** Distribution of the NORs between the emerging daughter HeLa cells in telophase

	0	1	2	3	4	ED	NED
Model	0.22	0.34	0.28	0.14	0.02	1.40	
Experiment	0.64	0.32	0.03	0.01	0	0.41	0.03

The table shows frequencies with which the number of rDNA FISH signals in the daughter cells differs by 0 (symmetrical distribution), 1, 2, and so on. The data, obtained from 200 cells, were compared with a random pairing model (see Section 2.3). In most cases NORs, in contrast to the competent NORs, were equally distributed between the daughter cells. The value of ED shows that in average daughter cells differed by less than one signal. Low value of NED emphasize the contrast with the case of competent NORs (Compare Tables 1 and 2).
